# A matter of child monitoring: a multistage analysis examining factors influencing parent use behavior of parental control apps

**DOI:** 10.3389/fpsyg.2026.1846156

**Published:** 2026-05-19

**Authors:** Ali Alkhalifah, Umar Ali Bukar

**Affiliations:** 1 Department of Information Technology, College of Computer, Qassim University, Buraidah, Saudi Arabia; 2 Department of Computer Science, Faculty of Computing and Artificial Intelligence, Taraba State University, Jalingo, Nigeria

**Keywords:** artificial neural networks, child monitoring, parental control apps, parental mediation, PLS predict, task-technology fit

## Abstract

**Introduction:**

The adoption and acceptance of parental control apps (PCAs) are threatened by many factors, even though mobile applications are at the forefront of mobile computing technologies. Existing studies indicate that PCA adoption can be improved by understanding users’ behavioral intention and mindsets. Several adoption studies show that task features are among the most influential factors influencing users’ perceptions of mobile applications. The role of PCA features, such as digital etiquette, cyberbullying, child tracking, and family values, remains largely unexamined in current research.

**Methods:**

Thus, by using task-technology fit (TTF) theory, this study examines how task-technology features and characteristics affect the behavioral intention of parents toward PCA adoption within the context of Saudi Arabia. To empirically test the proposed theoretical model, data were collected from 388 parents in Saudi Arabia. A multistage analysis, which consists of partial least squares structural equation modeling (PLS-SEM), partial least squares prediction algorithms (PLS-predict), and artificial neural networks (ANN), was employed to examine the outcome of PCA behavioral adoption.

**Results:**

The results demonstrate that digital etiquette, cyberbullying, family values, and parental mediation significantly and positively influence TTF, whereas mobility, child tracking, and inappropriate online content do not have a significant impact. Furthermore, TTF was found to be a significant predictor of parents’ behavioral intention toward PCA adoption. These findings can be utilized to enhance the design of PCAs and thus the user experience for both parents and children. Moreover, this study extends and contributes to the understanding of task features in the context of PCAs through the TTF model.

## 1 Introduction

The reliance of society on mobile services to carry out daily tasks has expanded dramatically over the previous decade, and children are part of this trend (Feal et al., 2020; Xin et al., 2025). Many of today’s children cannot fathom living without access to the Internet. The United Nations Children’s Fund (UNICEF) expresses concerns regarding the fact that children’s access to the Internet remains a highly personal and confidential issue (UNICEF, 2021). Many children tend to use these devices in the privacy of their bedrooms, often with minimal supervision (Alrusaini and Beyari, 2022).

To protect their children’s digital lives, some parents use parental control apps (PCAs) to monitor their children’s activities and restrict their access to certain features on their smartphones (Anderson, 2016; Feal et al., 2020; Xin et al., 2025). A typical PCA enables parents to filter, monitor, and restrict communications, material, system features, and the application’s execution (Wisniewski et al., 2017). Other applications provide parents with detailed information on their children’s phone usage, social interactions, and physical location. Anderson (2016) reported that research by a few researchers highlighted that there are fewer parents (16%) who employed parental controls on their children’s cell phones, compared with similar software placed on home PCs (Wisniewski et al., 2017). The utilization of PCA tools has declined in recent years, dropping from 28% in 2010 to 22% a decade later (Gnanasekaran and De Moor, 2025).

This downward trend in adoption suggests a critical research gap: a potential mismatch between the technical functionalities of PCA and the actual “tasks” parents need to perform to safeguard their children. Numerous studies indicate that existing solutions address only narrow technical aspects—such as time limitations and content restrictions—while neglecting broader parental needs, including the safeguarding of information and the promotion of independent thought (Alguliyev et al., 2022; Xin et al., 2025). However, there is a lack of research on the adoption and/or usage of PCA, which covers issues and factors such as child surveillance, cyberbullying, digital etiquette, inappropriate online content, safety awareness, and participative parental mediation to utilize limiting activities (Monteiro et al., 2017; Alrusaini and Beyari, 2022; Stoilova et al., 2024; Gnanasekaran and De Moor, 2025). Moreover, the question of whether these concerns will promote the use of PCAs from the viewpoint of parents has not been investigated. Several studies advocate for a more comprehensive incorporation of diverse parental styles, parental mediation strategies, and an increased emphasis on the overall user experience as potential remedies in this context (Wisniewski et al., 2017; Alguliyev et al., 2022; Alrusaini and Beyari, 2022; Stoilova et al., 2024; Gnanasekaran and De Moor, 2025; Xin et al., 2025). Furthermore, the existing body of research rarely accounts for the cultural and geographic nuances that shape parenting styles.

This study focuses on the context of Saudi Arabia, where digital transformation is rapid and family values play a central role in the social structure; understanding the “fit” between technology and parental tasks is crucial for increasing adoption rates (Alrusaini and Beyari, 2022). It focuses specifically on parents of school-aged children and adolescents (aged 6–18), as this demographic represents the peak period of personal device adoption and exposure to autonomous online risks (Pew Research Center, 2020). Hence, the aim of this study is to investigate factors influencing the adoption of PCA by examining task features and characteristics through the task-technology fit (TTF) model (Goodhue and Thompson, 1995). These factors include mobility, digital etiquette, cyberbullying, child tracking, parental mediation, control of inappropriate online content, and family values. The research question explored in this study is: *How do these specific task-technology features and characteristics affect the behavioral intention of parents toward PCA adoption?*

This study is organized as follows: Section 2 provides a review of the literature regarding PCA and justifies the conceptualization of the research model based on TTF. Section 3 introduces the conceptual model, followed by Section 4, which outlines the research methodology. Section 5 presents the empirical results, whereas Section 6 provides a detailed discussion of the findings. The theoretical and practical contributions are detailed in Section 7, and the limitations and directions for future research are discussed in Section 8. Finally, Section 9 concludes the study.

## 2 Literature review

### 2.1 PCA adoption studies

Alelyani et al. (2019) analyzed 29,272 evaluation reports of 52 different PCAs from the Google Play Store. The study discovered that parent-written reviews differ statistically from child-written reviews; these differences illuminate the complexities and tensions inherent in parent–child relationships as mediated by the usage of PCAs. Some studies investigated parental perceptions of their children’s adoption of mobile learning applications with in-app adverts (Hsu et al., 2020).

Ghosh et al. (2018) investigated how children perceive PCAs through a qualitative analysis of 736 publicly posted evaluations of 37 mobile online safety apps on Google Play by users aged 8–19. The findings reveal that child ratings are much lower than parent evaluations, and children perceived the applications to be excessively limiting and intrusive to their personal privacy, which had a detrimental effect on their relationships with their parents (Ghosh et al., 2018). Furthermore, Wisniewski et al. (2017) conducted a structured, qualitative analysis of 75 Android mobile apps intended to promote adolescent online safety. The results showed distinct characteristics that corresponded to a conceptual framework of teen Internet safety techniques that strike a compromise between parental control measures (monitoring, limitation, and active mediation) and teen self-regulation strategies (self-monitoring, impulse control, and risk-coping). The apps strongly valued parental control features such as monitoring and controlling kids’ online habits over teen self-regulation or more communicative and collaborative practices between parents and teens (Wisniewski et al., 2017).

### 2.2 Divergent perceptions: parent versus child perspectives

Monteiro et al. (2017) reviewed the use of mobile applications to promote online safety among children and youth. Interestingly, their study highlighted a paradox: although review papers on PCAs document poor adoption rates and advocate for broader alternative solutions, the persistent low adoption among parents serves as a primary motivation for exploring how these apps can effectively bridge safety gaps. Despite low adoption, research focusing on the development or utilization of specific apps addresses critical issues such as cyberbullying, digital etiquette, exposure to inappropriate content, safety awareness, participatory parental mediation, and child tracking (Monteiro et al., 2017). However, these factors have not been sufficiently examined to determine whether they truly encourage PCA adoption from a parental perspective, particularly within the context of Middle Eastern countries.

Further investigation by Gnanasekaran and De Moor (2025) explored the potential of next-generation parental controls to enhance children’s digital competence and cybersecurity awareness. Through six semi-structured interviews with parents (whose children were aged 10–13) and five domain experts, alongside four focus groups with pre-teens (*N* = 14), their findings revealed a significant gap: current parental control tools are often perceived as non-educational and lack the necessary features to improve children’s digital literacy or cybersecurity resilience.

Finally, Stoilova et al. (2024) conducted a systematic review of 1,656 articles concerning parental control tools. Their research indicated that the use of these tools is influenced by the ages of both parents and children, their respective digital competencies, levels of parental engagement, and a proactive desire to mitigate online risks. However, the outcomes of using such controls remain inconsistent; findings suggest that they can yield positive or negative effects, restrict certain developmental results, or produce no significant impact at all. Although the review provided limited evidence for promoting parental controls as a standalone strategy, it noted that parents value these tools most when they are integrated into a comprehensive framework of parental mediation and healthy parent–child relationships.

### 2.3 Limitations of existing PCA

Recent PCA studies suggest that current solutions remain narrow in scope, primarily focusing on technical barriers like time management and content filtering. However, these technologies frequently overlook critical dimensions of the digital experience, such as information security and the cultivation of children’s cognitive autonomy (Alguliyev et al., 2022; Xin et al., 2025). Furthermore, there is a distinct shortage of empirical research connecting the adoption of PCA to broader socio-behavioral issues. Factors such as the ethics of child surveillance, digital etiquette, cyberbullying, inappropriate online content, and the transition from restrictive oversight to participative parental mediation remain largely underexamined in the existing literature (Monteiro et al., 2017; Alrusaini and Beyari, 2022; Stoilova et al., 2024; Gnanasekaran and De Moor, 2025).

### 2.4 Task-technology fit model

The TTF model examines how the technological features of an information system align with a user’s existing tasks to enhance overall performance (Goodhue and Thompson, 1995; Gerhart et al., 2015; Howard and Rose, 2019). At the core of the TTF model is the assessment of how effectively integrated technologies meet the specific functional requirements of the user. TTF has been widely utilized in information systems research and is frequently integrated with other frameworks, such as social cognitive theory (Lin and Huang, 2008), the information systems success model (Tam and Oliveira, 2016b), and the unified theory of acceptance and use of technology (UTAUT) (Zhou et al., 2010). Furthermore, the specific context of use established by a technology is critical for its long-term acceptability and adoption (Mallat et al., 2009).

The TTF theory is particularly relevant to this research. A recent study by Chua et al. (2025) indicated that TTF plays a vital role in understanding technology adoption, revealing that higher levels of fit correlate with favorable user outcomes, such as increased satisfaction and acceptance. Consequently, it is essential to assess how well PCAs align with the lifestyles of children and the needs of their parents to determine the success of their adoption. The compatibility of PCA can be evaluated by examining both task and technology characteristics; prior research has established that high TTF significantly enhances user receptiveness (Li et al., 2019; Chua et al., 2025).

Given the success of TTF in predicting behavioral intentions for mobile applications (Tam and Oliveira, 2016a; Li et al., 2019; Alharbi and Alkhalifah, 2024), this study applies the model to better understand the intention to use PCAs. Drawing on previous TTF findings, this study proposes a conceptual model to establish the task-technology characteristics and use context of PCA, based on the assumption that task features significantly influence both the adoption and usage intentions of these applications.

## 3 Research model and hypothesis development

**Figure 1:**
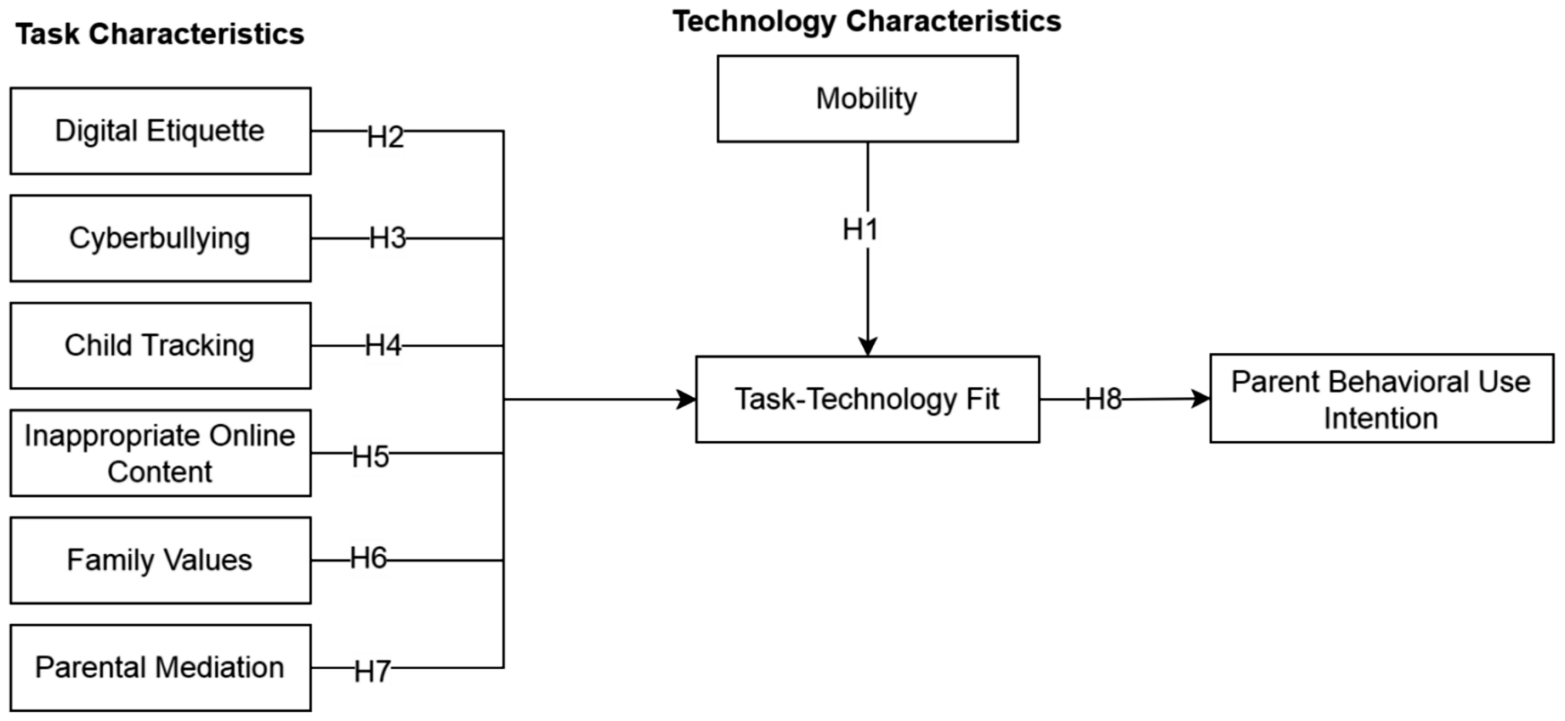
Conceptual model of parental control app adoption.

This study extends and contributes to the understanding of task features in the context of PCAs through the TTF model. Task characteristics are defined as the functional responsibilities and goal-oriented actions performed by parents to manage their children’s digital safety. These are not merely psychological states, but information-processing activities that require inputs (monitoring) and outputs (intervention/protection). These include digital etiquette, cyberbullying, family values, parental mediation, child tracking, and inappropriate online content. Technology characteristics refer to the specific features and capabilities inherent in the PCAs used to perform the tasks. In this research, these characteristics include mobility. The conceptual model and hypotheses are presented in Figure 1.

### 3.1 Mobility

PCAs are created due to the parents’ desire to control their children’s digital activities (Feal et al., 2020). The amount of research conducted on PCA features influencing usage intentions has not been substantial (Monteiro et al., 2017; Mannan et al., 2020; Stoilova et al., 2024). Hence, technology characteristics have been viewed as explicit technological components within the TTF framework (Kim et al., 2015; Mallat et al., 2009; Tam and Oliveira, 2016a; Tam and Oliveira, 2016b). In the TTF framework, technology characteristics are the features that enable a user to perform a specific task more effectively. This study identifies mobility as the foundational technology characteristic of PCA. Mobility is defined as the degree to which users can access and utilize the application regardless of time or location (Mallat et al., 2009; Zhou et al., 2010).

The theoretical rationale for its positive influence on TTF lies in the “matching” logic. The primary task of digital parenting—monitoring and protecting a child—is increasingly decentralized. As children move between school, social activities, and various outdoor environments, the parenting task is no longer bound to a physical home location (Monteiro et al., 2017). Consequently, there is a functional requirement for a “technical match” that operates with the same degree of ubiquity as the task itself.

If a PCA lacked mobility (e.g., if it worked only on a home Wi-Fi network), it would fail to “fit” the task of monitoring a child who is at school or the playground. Therefore, the fit is enhanced when the technology’s mobile capability mirrors the spatial flexibility required by the parent’s monitoring task. By allowing parents to perform parenting activities “anywhere and at any time,” mobility directly reduces the effort and increases the effectiveness of the intervention (Li et al., 2019). The mobility feature of PCA provides the necessary technical flexibility to match the location-independent nature of modern parenting tasks, thereby positively influencing the perceived TTF. Hence, we hypothesize:

*H1*: The mobility feature of parental control apps positively influences parents’ task-technology fit.

### 3.2 Digital etiquette

Digital etiquette is a foundational component of the digital citizenship framework, defined as the standards of conduct governing online participation and social interaction (Ribble, 2012; Wang and Xing, 2018). In the context of this study, digital etiquette is conceptualized as a critical “task characteristic” because it represents a functional parental obligation: the need to reinforce ethical online behavior and discourage risky social conduct in a child’s virtual life.

The theoretical rationale for the “fit” between digital etiquette and PCA technology lies in the complexity and invisibility of the task. Teaching etiquette in a physical setting relies on direct observation; however, in a digitally enhanced environment, a child’s social interactions are often hidden from the parent (Ribble and Bailey, 2004). This creates a “task-information gap” in which the parent is responsible for a behavior that they cannot see.

Within the TTF framework, the PCA serves as the technological match by providing visibility and oversight. When an application offers features—such as activity logs, keyword alerts, or social interaction reports—it provides the parent with the necessary “inputs” to fulfill the “task” of etiquette mediation (Abd Rahman et al., 2014; Monteiro et al., 2017; Singh et al., 2017). A high degree of fit occurs because the technology automates the monitoring of standards that are otherwise difficult for the parent to track manually. Therefore, if a parent perceives that the PCA effectively aids in managing the child’s digital conduct and social responsibilities, the perceived TTF will be enhanced (Abulibdeh, 2019; Wang and Xing, 2018). The digital etiquette features of PCAs provide a technical solution for monitoring invisible social conduct, thereby positively influencing the parents’ perceived TTF. Hence, we hypothesize:

*H2*: The digital etiquette feature of parental control apps positively influences the parents’ task-technology fit.

### 3.3 Cyberbullying

Cyberbullying represents a targeted online offense designed to inflict psychological and emotional distress, often falling into a unique threat category between security, safety, and privacy (Kshetri and Voas, 2019). Within this study’s framework, cyberbullying is defined as a high-stakes “task characteristic” for the parent. The parenting task involves the continuous monitoring of digital discourse to detect and mitigate social aggression directed at or perpetrated by the child.

The theoretical rationale for the “fit” between cyberbullying prevention and PCA technology is rooted in the cognitive and scale limitations of human oversight. Internet users, particularly “digital natives,” engage in vast amounts of social interaction across multiple platforms, often lacking the associated technological ethics (Zhong et al., 2021). For a parent, the manual task of reviewing thousands of messages or social media comments to identify subtle bullying patterns is functionally impossible. This creates a “task-processing gap” in which the volume and velocity of digital communication exceed the parent’s monitoring capacity.

Consequently, there is a fundamental requirement for a technological match that offers automated, real-time detection and alerting functionalities (Silva et al., 2016; Monteiro et al., 2017). When a PCA provides features like Facebook’s “BullyBlocker,” it directly “fits” the task by reducing the parents’ cognitive load and providing a proactive rather than reactive response to incidents. The fit is further enhanced when the technology aligns with the parents’ desire to cultivate digital citizenship awareness, effectively acting as a digital forensic tool to decipher hidden virtual threats (Kshetri and Voas, 2019). Thus, the more the technology can automate the identification of these social risks, the higher the perceived TTF. The cyberbullying features of PCAs provide a technical match for the high-volume monitoring tasks of parents, thereby positively influencing the perceived TTF. As a result, we hypothesize:

*H3*: The cyberbullying feature of parental control apps positively influences parents’ task-technology fit.

### 3.4 Child tracking

The task of child monitoring has fundamentally shifted as children gain greater physical mobility outside the home (Maslen, 2021). Within the TTF framework, the “task” is defined as the parental obligation to ensure the child’s physical safety when they are not in the parent’s immediate line of sight (e.g., traveling to school or attending social events) (Monteiro et al., 2017).

While existing literature highlights a “privacy-protection” paradox in which children may perceive tracking as intrusive (Gabriels, 2016), the TTF is evaluated from the user’s (parent’s) perspective. For a parent, the cognitive task of “worrying” or “verifying” a child’s location is high-effort and low-certainty. The technology matches this task by providing real-time geospatial data, which replaces parental uncertainty with automated verification.

The rationale for a positive hypothesis lies in the functional utility of the fit: when the parent’s task is “safety assurance,” a tracking feature that provides accurate location data reduces the parent’s mental workload (Sukk and Siibak, 2021). Thus, even if social tensions exist, the functional fit between the need for safety information and the provision of location data is theoretically positive. Hence, we hypothesize:

*H4*: The child tracking feature of parental control apps positively influences parents’ task-technology fit.

### 3.5 Inappropriate online content

The proliferation of digital platforms has significantly increased the probability of children encountering inappropriate content, including violent imagery or age-restricted content, which can endanger their psychological health (Cho and Cheon, 2005; Narayanan and Nirmala, 2018). Within the TTF framework, the “task” is defined as the parental obligation to curate a safe digital environment—a process analogous to “gatekeeping.” This task involves the constant evaluation of digital material to ensure that it aligns with the child’s developmental stage (Brito et al., 2017).

The theoretical rationale for the “fit” between this task and PCA technology lies in the information asymmetry between parents and children. Research indicates that youngsters are exposed to more unpleasant Internet information than most parents realize, creating a gap between the parents’ “perceived control” and the actual “digital reality” (Cho and Cheon, 2005). The manual task of content curation is hindered by the sheer volume of data and the private nature of mobile device usage.

Consequently, there is a functional requirement for a technological match that provides automated filtering, reporting, and blocking capabilities. A high degree of fit occurs when the PCA functions as a “technical gatekeeper,” enabling the co-regulatory approach encouraged in the literature (De Almeida et al., 2012; Monteiro et al., 2017). By providing real-time content analysis and removal tools, the technology reduces the parents’ monitoring effort while increasing the efficacy of the safety “task.” Therefore, the more effectively a PCA identifies and restricts inappropriate content, the more it satisfies the parent’s environmental control needs, leading to a higher perceived TTF. Hence, we hypothesize:

*H5*: The control of inappropriate online content features of parental control apps positively influences parents’ task-technology fit.

### 3.6 Family values

Family values—encompassing principles such as honesty, trust, and discipline—serve as the foundational framework for parenting strategies and adolescent development (Jagger and Wright, 1999; Wisniewski et al., 2017). Within the TTF model, the “task” is defined as the parental obligation to align digital behavior with the family’s specific moral and social codes. This is a complex, context-dependent task that varies based on socioeconomic status and chosen parenting styles, such as authoritarian, indulgent, or authoritative approaches (Erickson et al., 2016).

The theoretical rationale for the “fit” between family values and PCA technology lies in the need for value-sensitive design (VSD). Traditional safety applications often “misfit” the parenting task because they impose rigid control mechanisms that prioritize monitoring over trust-building (Badillo-Urquiola et al., 2020). For a parent whose “task” is to foster autonomy and self-regulation (authoritative parenting), a technology that only allows for restrictive blocking creates a functional conflict.

Consequently, there is a requirement for a technological match that offers customizable features reflecting a family’s unique value system (Czeskis et al., 2010). A high degree of fit occurs when the PCA provides features that support transparency and collaborative oversight rather than just unilateral surveillance. By incorporating “positive” family values—such as trust-based reporting or shared safety agreements—into the app’s design, the technology directly assists the parent in the task of moral guidance. Therefore, the more a PCA allows parents to mirror their offline parenting values in the digital sphere, the higher the perceived TTF. As a result, we hypothesize:

*H6*: The family value feature in parental control apps positively influences parents’ task-technology fit.

### 3.7 Parental mediation

Parental mediation is defined as the strategies parents employ to manage and regulate their children’s digital experiences, traditionally categorized into restrictive, active, and co-viewing mediation (Stoilova et al., 2024; Baumrind, 2005). Within the TTF model, parental mediation is conceptualized as a “task characteristic” because it represents the functional process of negotiating boundaries and safety rules between parent and child. Unlike purely technical monitoring, this task involves an evolving balance between parental authority and the child’s developing autonomy as they mature (Gnanasekaran and De Moor, 2025).

The theoretical rationale for the “fit” between parental mediation and PCA technology lies in the asymmetry and social tension inherent in digital parenting. Research highlights a significant disparity in which current technologies heavily favor restrictive monitoring over adolescent self-regulation (Wisniewski et al., 2017). This creates a task-misfit for parents who wish to move beyond fear-based tactics toward a more collaborative or authoritative mediation style (Baumrind, 2005).

Consequently, there is a functional requirement for a technological match that provides interactive features, such as shared time-limit agreements, request-and-approve mechanisms, and age-appropriate configuration settings (Dias and Brito, 2020). A high degree of fit occurs when the PCA functions as a communication facilitator rather than just a surveillance tool. By offering features that allow for the “collaborative setting of limitations” across different age groups (under 12, 13–15, and 16–17), the technology directly supports the parent’s mediation task. Therefore, the more a PCA enables a parent to balance protection with the child’s need for privacy and autonomy, the higher the perceived TTF. Hence, we hypothesize:

*H7*: The parental mediation feature in parental control apps positively influences parents’ task-technology fit.

### 3.8 Task-technology fit

According to the TTF framework, the primary driver of technology adoption is not the technology’s sophistication itself, but how effectively it assists the user in completing a specific task (Goodhue and Thompson, 1995; Zhou et al., 2010). In this research, behavioral intention is the parents’ psychological readiness to adopt a PCA. When a parent perceives a high “fit,” they recognize that the application successfully automates or simplifies the complex tasks of digital parenting (e.g., location tracking, content filtering, and social monitoring). This perceived reduction in cognitive load and the corresponding increase in parental efficacy create a positive attitude toward the system. Literature across various domains—including MOOCs, emergency response, and mobile banking—consistently shows that TTF is a dominant predictor of adoption (Tam and Oliveira, 2016a; Li et al., 2019; Alkhalifah and Bukar, 2022). For parents, if the PCA is perceived as a “perfect match” for their duty of care, their intention to use the app increases because the technology is observed as a necessary partner in their parenting strategy rather than an intrusive or redundant tool. Hence, we hypothesize:

*H8*: The task-technology fit positively influences parents’ behavioral intention.

## 4 Methodology

### 4.1 Sampling and data collection procedure

The Kingdom of Saudi Arabia (KSA) was selected as the research context for several reasons. First, KSA has one of the highest smartphone penetration rates globally, with a rapidly digitizing education sector that increases the functional necessity of PCAs (Alrusaini and Beyari, 2022). Second, recent government initiatives in KSA have advocated for digital safety and cybersecurity awareness, providing a timely environment to study adoption drivers. Finally, there is a recognized lack of empirical research focusing on parental control adoption within Middle Eastern and Asian demographics (Xin et al., 2025; Stoilova et al., 2024), making this study a critical contribution to the global understanding of digital parenting.

This study utilized a non-probability intercept sampling method (Memon et al., 2025). Data collection was conducted at multiple high-traffic city malls to reach a diverse cross-section of the target population. Researchers approached individuals and couples accompanied by children at random intervals. To minimize selection bias, the intercepts occurred at varying times of day across both weekdays and weekends. A barcode was generated with the study description and survey link. The barcode was presented to the target participants. Participants were screened based on a set of inclusion criteria: they were required to be a legal parent of at least one child (under 18 years of age), a resident of KSA, and familiar with the basic concept or existence of parental control services.

The study was conducted in accordance with the ethical standards and received formal approval from the Ethics Committee of Qassim University. Prior to participation, all respondents were provided with a clear study description outlining the research objectives. Informed consent was obtained digitally before the survey commenced.

Following data collection and cleaning, 388 valid responses constituted the final study sample. This sample size fulfills the requirements for conducting partial least squares (PLS) structural equation modeling and confirmatory factor analysis (Chin, 1998; Goodhue et al., 2012; Hair et al., 2017a; Hair et al., 2017b; Hair et al., 2021). Regarding participant demographics, 62.6% were women and 37.4% were men, with 73.2% of the sample aged over 30 years. Additionally, a majority of the participants (69%) reported having more than one child, and 65.9% held at least a bachelor’s degree.

### 4.2 Measurement scales and instrument development

To ensure content validity, the measurement items for this study were adapted from established scales in the existing literature and modified to align with the specific context of PCAs (Churchill, 1979). Specifically, items for mobility were adapted from Mallat et al. (2009), Zhou et al. (2010), and Li et al. (2019). Measures for child tracking were derived from Sukk and Siibak (2021), whereas cyberbullying scales were adapted from Zheng et al. (2020) and Zhong et al. (2021). Digital etiquette items were obtained from Abulibdeh (2019), and family value measurements were based on the work of Wisniewski et al. (2017) and Badillo-Urquiola et al. (2020). Furthermore, items regarding inappropriate online content were sourced from Brito et al. (2017), and parental mediation scales were adapted from Ko et al. (2015) and Monteiro et al. (2017). Finally, the TTF constructs were obtained from Zhou et al. (2010) and Li et al. (2019), whereas behavioral intention was measured using scales from Venkatesh et al. (2003).

Regarding the composition of the instrument, Hinkin (1998) noted that although no rigid rule dictates the number of items per construct, adequate sampling of each variable is essential. Following the recommendation of Gefen et al. (2011), each variable in this study was measured using at least three indicators to ensure statistical identification (see Appendix A). All items were evaluated using a five-point Likert scale, ranging from “1” (Strongly Disagree) to “5” (Strongly Agree).

To further refine the instrument, a pilot study was conducted to ensure face and content validity (Mackenzie et al., 2011; Hair et al., 2021). The questionnaire was reviewed by a panel consisting of five information systems professors and 10 parents who actively use PCA services. Based on their feedback and expert comments, minor adjustments were made to the wording of several items to improve clarity and contextual relevance.

### 4.3 Data analysis

The research model was tested using SmartPLS 4, employing the PLS structural equation modeling method (Hair et al., 2017a, 2017b). Confidence intervals were computed using the bootstrapping procedure with 10,000 sub-samples (Streukens and Leroi-Werelds, 2016). Beyond traditional model fit indices, the PLS-predict algorithm was utilized to evaluate the model’s out-of-sample predictive relevance (Shmueli et al., 2016).

Subsequently, an artificial neural network (ANN) analysis was applied to re-examine the model’s predictive capacity. The ANN is a non-linear modeling tool capable of replicating human neural systems to learn and predict complex behavioral patterns (Chong, 2013). By training its learning capacities, the performance and accuracy of these networks can be significantly enhanced (Chang et al., 2012; Teo et al., 2015). The ANN analysis was conducted using IBM SPSS v24, following established methodologies in recent literature (Hew et al., 2018; Li et al., 2019; Alkhalifah and Bukar, 2022; Alharbi and Alkhalifah, 2024, 2025).

Specifically, a multi-layer perceptron with a feed-forward back-propagation algorithm was employed to estimate the relative importance of the independent variables. To ensure robust results and minimize overfitting, a 10-fold cross-validation procedure was performed. For each of the 10 resulting ANN models, the dataset was partitioned into 70% for training and 30% for testing to assess the network’s predictive accuracy.

A hybrid SEM-ANN approach was adopted to bridge the gap between theoretical explanation and predictive accuracy. While PLS-SEM and PLS-predict are essential for testing hypothesized linear relationships and out-of-sample relevance, they often fail to capture the non-linear, non-compensatory complexities inherent in human decision-making (Hew et al., 2018; Li et al., 2019). By integrating ANN, this study overcomes the linearity constraints of SEM, utilizing its predictive power to validate the importance of predictors through sensitivity analysis (Alkhalifah and Bukar, 2022). This two-stage framework ensures that the model is not only theoretically sound but also practically robust in predicting real-world adoption behavior (Alharbi and Alkhalifah, 2024).

## 5 Results

Before testing the hypothesized relationships, the data were subjected to several diagnostic tests to ensure suitability and the absence of bias. First, Harman’s single-factor test was used to assess common method bias (CMB) (Harman, 1976). The analysis revealed that a single factor accounted for only 30.12% of the total variance. As this result is well below the 50% threshold recommended in the literature (Harman, 1976; Podsakoff et al., 2003), CMB is not a significant concern in this study.

Second, Bartlett’s test of sphericity was conducted to evaluate the correlation matrix of the research items. The results showed that the significance level for this study was 0.00, indicating that the variables are sufficiently correlated for further multivariate analysis. Additionally, the Kaiser–Meyer–Olkin (KMO) measure of sampling adequacy resulted in a value of 0.895. This score is categorized as “meritorious” or “marvelous,” confirming that the sample is highly adequate for factor analysis. Consequently, the results of these diagnostic tests confirm that the dataset is robust, free from significant bias, and appropriate for structural equation modeling.

### 5.1 Measurement model assessment

The data were assessed for normality issues, and the result was satisfactory. Next, the data were subjected to a reliability test, which was determined using convergent validity techniques. According to the result of convergent validity, all item loadings exceed 0.600, more precisely ranging between 0.614 and 0.871, as indicated in Table 1. These results demonstrate that the items and their constructs display a sufficient degree of variance (Chin, 1998; Hair et al., 2019; Hair et al., 2021). However, a few items (CTR3, TTF1, and TTF2) had loading values less than 0.700. Nevertheless, these items were retained for further analysis because the average variance extracted (AVE) values are acceptable (Hair et al., 2017a). Hence, the reliability metrics for the model’s latent variables are summarized in Table 1. Furthermore, the Cronbach’s alpha values range from 0.704 to 0.816 and are >0.70, indicating strong item reliability. Additionally, for composite reliability to be demonstrated, the rho_A coefficient should be >0.7, which in this study ranges from 0.707 to 0.818. In a similar vein, the composite reliability values are higher than 0.700, with values ranging from 0.710 to 0.891. Similarly, all AVE metrics are >0.500, with values ranging from 0.538 to 0.731, respectively (Chin, 1998; Hair et al., 2019; Hair et al., 2021). Hence, these statistical measurement indices show that the measurement model accurately represents the data.

**Table 1:** Item loadings and reliability measures.

Construct	Items	Loadings	Cronbach’s alpha	rho_A	Composite reliability	AVE
	BI1	0.780	0.722	0.732	0.843	0.641
	BI2	0.785				
	BI3	0.836				
	CBB1	0.712	0.766	0.787	0.775	0.538
	CBB2	0.737				
	CBB3	0.788				
	CTR1	0.796	0.704	0.709	0.790	0.557
	CTR2	0.776				
	CTR3	0.614				
	DET1	0.829	0.734	0.748	0.848	0.651
	DET2	0.832				
	DET3	0.757				
	FAV1	0.800	0.737	0.744	0.804	0.578
	FAV2	0.747				
	FAV3	0.733				
	IOC1	0.821	0.707	0.711	0.837	0.632
	IOC2	0.830				
	IOC3	0.730				
	MOB1	0.833	0.816	0.818	0.891	0.731
	MOB2	0.871				
	MOB3	0.860				
	PAM1	0.712	0.747	0.762	0.806	0.582
	PAM2	0.744				
	PAM3	0.829				
	TTF1	0.697	0.770	0.756	0.710	0.553
	TTF2	0.621				
	TTF3	0.786				

The discriminant validity of the measures was estimated by comparing the indicator loadings of the constructs with those of the other constructs. As presented in Table 2, the results demonstrate that all indicators loaded at or above the loading of the other constructs. Similarly, the loadings of each indicator on its construct were significantly greater than the loadings of the indicators on the other variables in the sample, which shows a significant level of discriminant validity. Apart from analyzing the cross loadings, the Fornell–Larcker criterion was used to determine the discriminant validity of the measurement model, as shown in Table 3. Accordingly, two criteria must be met to establish discriminant validity based on the Fornell–Larcker index (Fornell and Larcker, 1981). First, the square root of each construct’s AVE must be greater than its correlation with another construct. Second, each item must load disproportionately higher on the construct with which it is most closely correlated (Chin, 1998; Henseler et al., 2015). Similarly, according to the Fornell–Lacker result, all values are statistically significantly larger than the correlated constructs, indicating that the discriminant validity of the measurement model is acceptable.

**Table 2:** Crossloadings of measurement items.

Construct	BI	CTR	CBB	DET	FAV	IOC	MOB	PAM	TTF
BI1	0.780	0.189	0.259	0.277	0.262	0.245	0.516	0.413	0.222
BI2	0.785	0.365	0.319	0.302	0.359	0.333	0.551	0.462	0.181
BI3	0.836	0.417	0.442	0.293	0.414	0.489	0.542	0.549	0.241
CTR1	0.349	0.796	0.561	0.326	0.348	0.434	0.376	0.350	0.255
CTR2	0.264	0.776	0.372	0.268	0.341	0.431	0.288	0.299	0.244
CTR3	0.274	0.614	0.363	0.255	0.188	0.296	0.163	0.261	0.187
CBB1	0.394	0.459	0.712	0.252	0.373	0.480	0.364	0.384	0.297
CBB2	0.277	0.385	0.737	0.278	0.357	0.482	0.248	0.309	0.268
CBB3	0.291	0.481	0.788	0.394	0.332	0.382	0.283	0.328	0.336
DET1	0.323	0.289	0.360	0.829	0.273	0.277	0.287	0.322	0.417
DET2	0.270	0.335	0.320	0.832	0.304	0.287	0.330	0.365	0.429
DET3	0.285	0.317	0.338	0.757	0.331	0.287	0.212	0.273	0.316
FAV1	0.348	0.282	0.360	0.247	0.800	0.439	0.283	0.426	0.168
FAV2	0.261	0.339	0.353	0.318	0.747	0.426	0.233	0.322	0.135
FAV3	0.371	0.320	0.366	0.290	0.733	0.416	0.357	0.524	0.144
IOC1	0.329	0.461	0.476	0.258	0.313	0.821	0.254	0.405	0.257
IOC2	0.353	0.428	0.533	0.238	0.402	0.830	0.356	0.416	0.253
IOC3	0.394	0.385	0.403	0.344	0.639	0.730	0.369	0.459	0.234
MOB1	0.523	0.339	0.293	0.273	0.334	0.318	0.833	0.572	0.179
MOB2	0.574	0.353	0.393	0.349	0.336	0.394	0.871	0.516	0.183
MOB3	0.617	0.299	0.338	0.268	0.309	0.332	0.860	0.507	0.163
PAM1	0.371	0.381	0.422	0.315	0.559	0.452	0.374	0.712	0.250
PAM2	0.540	0.227	0.271	0.295	0.368	0.362	0.540	0.744	0.177
PAM3	0.479	0.322	0.336	0.307	0.353	0.404	0.529	0.829	0.290
TTF1	0.235	0.076	0.225	0.140	0.138	0.152	0.192	0.245	0.697
TTF2	0.140	0.188	0.255	0.147	0.055	0.186	0.089	0.174	0.621
TTF3	0.190	0.307	0.325	0.537	0.179	0.267	0.145	0.241	0.786

Values in colour represent the primary factor loadings, indicating that the item loads more strongly on its intended construct than on any other construct (cross-loadings).

**Table 3:** Fornell–Larcker criterion matrix.

Construct	BI	CTR	CBB	DET	FAV	IOC	MOB	PAM	TTF
Behavioral intention	0.801								
Child tracking	0.403	0.733							
Cyberbullying	0.429	0.595	0.746						
Digital etiquette	0.362	0.387	0.418	0.807					
Family values	0.432	0.409	0.472	0.370	0.761				
Inappropriate online content	0.450	0.535	0.594	0.350	0.561	0.795			
Mobility	0.667	0.388	0.400	0.349	0.383	0.408	0.855		
Parental mediation	0.595	0.416	0.456	0.400	0.559	0.535	0.623	0.763	
Task-technology fit	0.271	0.314	0.405	0.486	0.197	0.312	0.205	0.323	0.673

### 5.2 Structural assessment model and hypothesis testing

**Figure 2:**
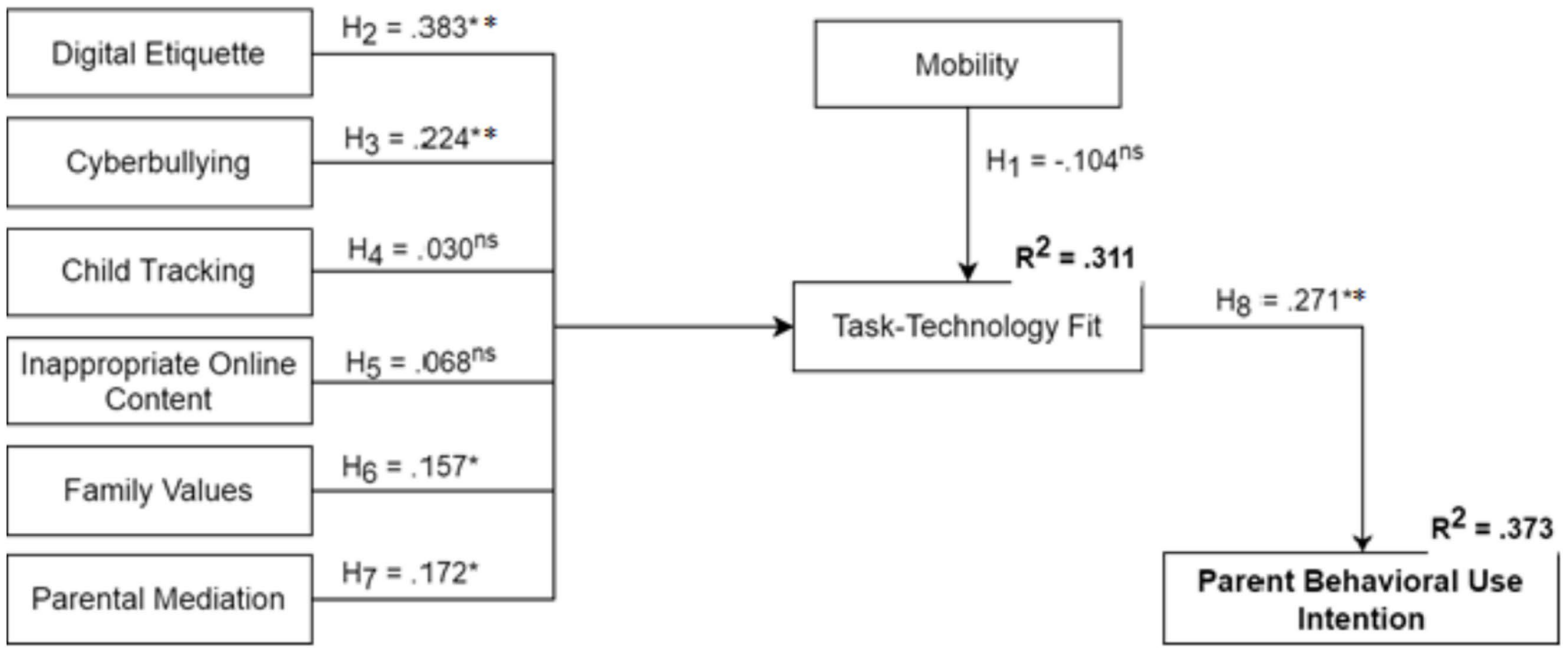
Structural model of PCA behavioral intention. n.s., non-significant; *p < 0.05; **p < 0.001 = significant.

The results of the hypothesized research relationships are illustrated in Figure 2 and further detailed in Tables 4, 5. The hypothesized relationships were assessed through path coefficients, which are presented in Table 4. Table 4 also contains the results for effect sizes (f^2^) and t-values, providing a comprehensive statistical evaluation of the hypotheses. Before assessing the structural model, multicollinearity was examined using the variance inflation factor (VIF). The results indicate that all VIF values are significantly below the threshold of 5, suggesting that collinearity does not pose a threat to the study’s findings (Hair et al., 2019). Notably, these results further validate the data robustness established by the previous CMB, KMO, and Bartlett’s tests. Consequently, it is appropriate to proceed with the discussion of the research hypotheses.

**Table 4:** PLS structural model results.

Relationships	*β*	SD	*T*-values	*p*-values	*f* ^2^	VIF
Mobility → task-technology fit	−0.104	0.066	1.571	0.116	0.009	1.715
Digital etiquette → task-technology fit	0.383	0.064	5.960	0.000	0.157	1.351
Cyberbullying → task-technology fit	0.224	0.069	3.235	0.001	0.037	1.979
Child tracking → task-technology fit	0.030	0.055	0.541	0.589	0.001	1.752
Inappropriate online content → task-technology fit	0.068	0.067	1.017	0.309	0.003	2.028
Family values → task-technology fit	0.157	0.065	2.403	0.016	0.020	1.750
Parental mediation → task-technology fit	0.172	0.075	2.303	0.021	0.020	2.184
Task-technology fit → behavioral intention	0.271	0.058	4.630	0.000	0.079	1.000

**Table 5:** Summary of hypothesis tests.

Code	Hypothesis	Supported
H1	The mobility feature of parental control apps positively influences parents’ task-technology fit	Rejected
H2	The digital etiquette feature of parental control apps positively influences parents’ task-technology fit	Accepted
H3	The cyberbullying feature of parental control apps positively influences parents’ task-technology fit	Accepted
H4	The child tracking feature of parental control apps positively influences parents’ task-technology fit	Rejected
H5	The control of inappropriate online content feature of parental control apps positively influences parents’ task-technology fit	Rejected
H6	The family value feature in parental control apps positively influences parents’ task-technology fit	Accepted
H7	The parental mediation feature in parental control apps positively influences parents’ task-technology fit	Accepted
H8	The task-technology fit positively influences parents’ behavioral intention	Accepted

The results of the hypothesis testing are summarized as follows: *H1* was not supported, as the relationship between task mobility and TTF was non-significant (*t* = 1.571, *p* = 0.116). Conversely, *H2* was supported, showing a strong positive relationship between digital etiquette and TTF (*t* = 5.960, *p* < 0.001). *H3* was also supported, confirming that cyberbullying significantly impacts TTF (*t* = 3.235, *p* < 0.01). However, the findings did not support *H4* and *H5*; neither child tracking (*t* = 0.541, *p* = 0.589) nor inappropriate online content (*t* = 1.017, *p* = 0.309) had a significant impact on TTF. In contrast, the influence of family values (*H6*) and parental mediation (*H7*) on TTF was positive and significant (*t* = 2.403, *p* < 0.05; *t* = 2.303, *p* < 0.05, respectively). Finally, the relationship between TTF and behavioral intention was significant (*t* = 4.630, *p* < 0.001), providing strong support for *H8*.

In structural equation modeling, the coefficient of determination (*R*^2^) represents the model’s explanatory power, where values provide insight into the variance explained in the endogenous constructs. Additionally, the predictive relevance (*Q*^2^)—obtained via the blindfolding algorithm—is typically expected to be lower than the *R*^2^ values (Hair et al., 2019, 2021). The findings indicate that the research model accounted for substantial variance in TTF (*R*^2^ = 0.311) and parental behavioral intention (*R*^2^ = 0.373). Although these values fall within the low-to-moderate range, they are considered substantial within the context of behavioral and social science research (Henseler et al., 2015; Hair et al., 2021). The *R*^2^ of 0.373 indicates that the model explains approximately 37% of the variance in a parent’s intention to adopt PCAs. Given that technology adoption is influenced by a vast array of external factors—including socioeconomic status, technical literacy, and varied parenting styles—explaining over one-third of the variance using a parsimonious set of predictors is statistically significant. Furthermore, the model yielded positive predictive relevance for both TTF (*Q*^2^ = 0.212) and behavioral intention (*Q*^2^ = 0.241). These results confirm that the structural model possesses sufficient explanatory power and predictive relevance, with the values for behavioral intention exceeding those for TTF.

### 5.3 PLS-predict

Researchers are urged to do additional analysis of the model by applying the PLS-predict approach, rather than simply reporting model fit, as is currently done (Shmueli et al., 2016). In this case, PLS-predict is a collection of procedures for the prediction of dependent variables that make use of PLS path models, as well as a measure of how predictable the model could be. The application of this method is due to the extensive and rapid advancement that has occurred in the PLS-SEM research field in recent years (Hamakhan, 2020). The method helps determine whether or not the model has predictive capabilities (Shmueli et al., 2019; Evermann and Tate, 2016). Nevertheless, it is advised that the measurement models used must meet the required criteria before initiating the PLS-predict approach.

Researchers are increasingly encouraged to conduct additional model analysis using the PLS-predict approach rather than relying solely on model fit indices (Shmueli et al., 2016). PLS-predict is a sophisticated procedure used to evaluate a model’s out-of-sample predictive power. Its adoption is driven by the rapid advancements in PLS-SEM research, emphasizing the importance of a model’s predictive capabilities (Shmueli et al., 2019; Hamakhan, 2020). However, it is essential that the measurement models meet all criteria for reliability and validity before implementing this approach. Given that the measurement model in this study demonstrated satisfactory reliability and validity, the PLS-predict algorithm was applied using a 10-fold cross-validation procedure.

Predictive relevance was assessed by comparing the root mean square error (RMSE), mean absolute error (MAE), and *Q*^2^_predict values of the PLS-SEM model against a naive linear model (LM) benchmark. Following the interpretation guidelines of Shmueli et al. (2019), the *Q*^2^_predict values are evaluated first; positive values indicate that the PLS-SEM model outperforms the LM benchmark. Subsequently, the prediction errors (RMSE and MAE) are compared. The PLS-predict algorithm was implemented by 10-fold cross-validation on the collected data. According to the results presented in Table 6, although the *Q*^2^_predict values were positive, the PLS-SEM model yielded fewer prediction errors for only a minority of the indicators compared with the LM. Therefore, following the criteria established by Shmueli et al. (2019), the research model is classified as having weak predictive power. This suggests that the relationships within the model may be complex and non-linear, necessitating further evaluation through a non-linear analytical tool such as an ANN to capture predictive patterns that traditional linear models may overlook.

**Table 6:** PLS-predict assessment of the original model (PLS) versus the naive benchmark (LM).

Item	PLS			LM			PLS-LM		
	RMSE	MAE	*Q*^2^_predict	RMSE	MAE	*Q*^2^_predict	RMSE	MAE	*Q*^2^_predict
BI3	0.735	0.531	0.087	0.622	0.443	0.346	0.113	0.088	−0.259
BI2	0.683	0.474	0.067	0.604	0.445	0.270	0.079	0.029	−0.203
BI1	0.917	0.706	0.054	0.797	0.611	0.285	0.120	0.096	−0.231
TTF2	0.942	0.709	0.037	0.964	0.732	−0.009	−0.022	−0.023	0.046
TTF3	0.605	0.460	0.259	0.608	0.436	0.252	−0.003	0.024	0.007
TTF1	0.782	0.604	0.012	0.778	0.599	0.023	0.004	0.006	−0.011

### 5.4 ANN analysis

**Figure 3:**
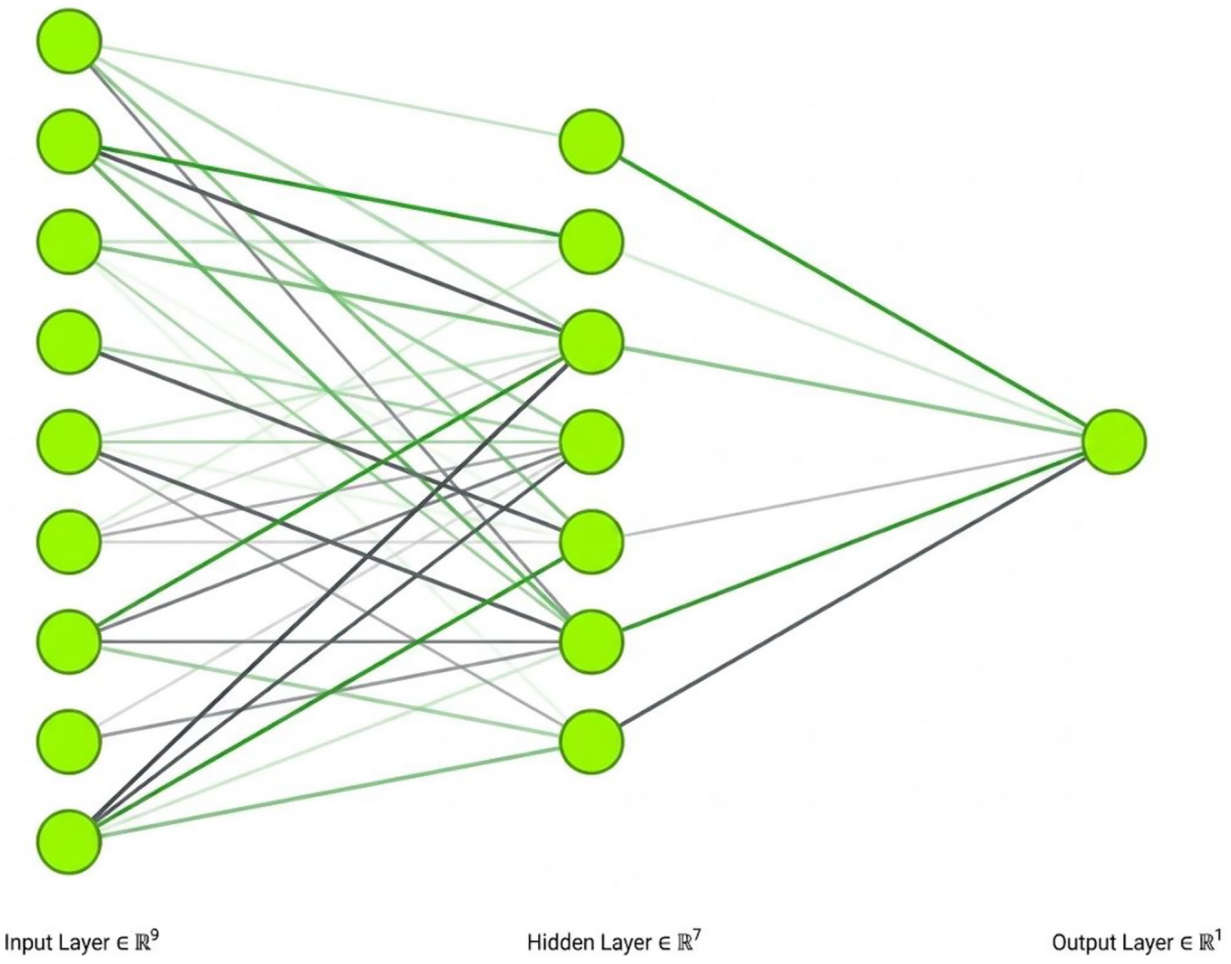
ANN model. Hidden layer activation function: hyperbolic tangent; output layer activation function: sigmoid; input neurons: child tracking, cyberbullying, digital etiquette, family values, inappropriate online content, mobility, parental mediation, and task-technology fit.

To further explore the model’s predictive relevance beyond linear constraints, an ANN analysis was conducted. The network architecture utilized a hidden layer with a hyperbolic tangent activation function, while the output layer was triggered using a sigmoid activation function. Figure 3 depicts a representation of the ANN model produced for one of the 10-fold cross-validation procedures using Nail (2019). To assess predictive accuracy, the RMSE was calculated for both the training and testing datasets across 10 folds (Hew et al., 2017; Li et al., 2019; Alharbi and Alkhalifah, 2025).

As shown in Table 7, the ANN model yielded an average RMSE of 0.485 for training and 0.499 for testing. These consistently low and similar RMSE values across both sets indicate that the model is robust and free from significant overfitting. Although the previous PLS-predict results suggested weak linear predictive power, the ANN results demonstrate a robust non-linear predictive capacity, confirming that the predictors are effective when non-linear complexities are accounted for.

**Table 7:** Root-mean-square error (RMSE) values for training and testing.

Network	Input neurons: child tracking, cyberbullying, digital etiquette, family values, inappropriate online content, mobility, parental mediation, and task-technology fit	
	Training	Testing
1	0.503	0.584
2	0.480	0.458
3	0.501	0.475
4	0.479	0.565
5	0.478	0.489
6	0.527	0.505
7	0.453	0.469
8	0.457	0.486
9	0.489	0.456
10	0.468	0.503
Mean	0.485	0.499
SD	0.023	0.046

SD, standard deviation.

To determine the contribution of each predictor, a sensitivity analysis was performed (Teo et al., 2015; Ooi and Tan, 2016). The relative importance and normalized importance of each exogenous variable are presented in Table 8. Interestingly, the non-linear analysis revealed that task mobility is the most influential predictor, with a normalized importance of 100%. This was followed by parental mediation (40.42%) and family values (31.21%). Other factors, including inappropriate online content (27.23%), child tracking (21.54%), digital etiquette (19.48%), TTF (19.11%), and cyberbullying (17.22%), contributed to a lesser degree. These findings suggest that although some variables may have weak linear influence, they remain critical components of the parental decision-making process in a non-linear context.

**Table 8:** Sensitivity analysis.

Network	Input neurons: child tracking, cyberbullying, digital etiquette, family values, inappropriate online content, mobility, parental mediation, and task-technology fit							
	TTF	DET	CBB	CTR	IOC	FAV	PAM	MOB
1	0.087	0.09	0.083	0.059	0.116	0.134	0.096	0.336
2	0.043	0.118	0.064	0.048	0.109	0.076	0.215	0.327
3	0.066	0.033	0.061	0.126	0.058	0.089	0.211	0.355
4	0.064	0.091	0.042	0.084	0.097	0.091	0.165	0.366
5	0.106	0.057	0.032	0.069	0.089	0.108	0.157	0.382
6	0.047	0.057	0.054	0.041	0.153	0.118	0.138	0.391
7	0.080	0.094	0.075	0.092	0.082	0.118	0.107	0.352
8	0.059	0.035	0.079	0.091	0.057	0.151	0.135	0.393
9	0.062	0.033	0.034	0.076	0.103	0.139	0.153	0.398
10	0.076	0.082	0.091	0.094	0.123	0.112	0.088	0.335
Mean (AVG)	0.069	0.069	0.0615	0.078	0.0987	0.1136	0.1465	0.3635
Normalized RI % (AVG)	19.11	19.48	17.22	21.54	27.33	31.21	40.42	100

*RI = relative importance.

## 6 Discussion

The purpose of this study was to investigate the features and characteristics that influence the adoption of PCAs by extending the TTF model. In this context, adoption refers to a parent’s capacity to successfully integrate and utilize PCA services within their daily routines. Consequently, this study examines the impact of fundamental task characteristics on both TTF and the behavioral intention to adopt PCAs. Following the multistage analysis framework established in prior research (Alharbi and Alkhalifah, 2025), this study utilizes a hybrid approach involving PLS-SEM, PLS-predict, and ANNs. This methodology was implemented to determine how effectively these characteristics forecast technology fit and drive adoption. The empirical results from these analyses are provided and discussed to validate the proposed research model.

Building upon literature regarding the potential for PCAs to strengthen parental monitoring, this study provides a necessary empirical assessment of parental behavior. The core objective is to advance the conceptualization of the TTF model, establishing how newly developed technological solutions align with a user’s existing tasks. This is particularly relevant as PCAs remain an emerging field in child monitoring (Xin et al., 2025; Stoilova et al., 2024; Gnanasekaran and De Moor, 2025).

The research findings revealed that five hypotheses were supported, whereas three were rejected. These results suggest that by actively developing solutions that address specific parental concerns, the adoption rate of these applications can be significantly enhanced. However, certain task features demonstrated no significant linear effect on TTF or overall behavioral intention. Specifically, although digital etiquette, cyberbullying, parental mediation, and TTF were statistically significant, mobility, control of inappropriate online content, and family values did not yield significant linear paths in the SEM analysis.

The significance of digital etiquette, cyberbullying, and parental mediation indicates that parents prioritize technologies that facilitate “active mediation” and protect a child’s psychological wellbeing within complex digital social structures (Stoilova et al., 2024). Conversely, the non-significance of mobility and content control suggests that these factors have become “baseline expectations” rather than active drivers of fit in a ubiquitous mobile environment (Gnanasekaran and De Moor, 2025). Furthermore, although family values were non-significant in the linear SEM, the robust link between TTF and behavioral intention confirms that parents adopt PCAs based on a utilitarian alignment between technical features and high-stakes parenting tasks (Xin et al., 2025). This theoretical synthesis moves beyond binary reporting to highlight how the “fit” of modern technological solutions is increasingly defined by social-psychological mediation rather than mere technical filtering.

The structural model’s explanatory power (*R*^2^ = 0.373 for behavioral intention) must be interpreted through the lens of the specific drivers identified in the hypothesis testing. Interestingly, the model reveals that parental adoption is driven more by digital etiquette and cyberbullying than by functional tools like child tracking or inappropriate content control. The lack of significance for H4 and H5 suggests that for this educated Saudi demographic, the “fit” of a PCA is not defined by basic surveillance, but by the management of complex social interactions. Furthermore, the non-significance of mobility indicates that parents may view PCA tasks as stationary or secondary to active supervision, rather than mobile-dependent. The moderate *R*^2^ is therefore a reflection of a highly selective adoption psychology in which only specific socio-technical factors—namely family values, mediation, and social threats—contribute to the variance. In behavioral research, an *R*^2^ of this magnitude is considered acceptable when identifying specific significant predictors within a complex social phenomenon (Hair et al., 2021).

The ANN analysis revealed a low average RMSE, indicating that the model possesses a robust non-linear predictive capacity (Alharbi and Alkhalifah, 2025). However, a notable divergence emerged: the sensitivity analysis identified mobility as the most influential predictor (100% normalized importance), followed by parental mediation and family values. This initially appears to contradict the PLS-SEM path coefficients, which showed mobility, inappropriate online content, and family values as statistically non-significant. Rather than a contradiction, this disagreement reveals a non-linear complexity that traditional linear models are unable to detect. Although these variables do not follow a constant, proportional “straight-line” relationship with adoption—leading to their non-significance in SEM—the ANN identifies them as non-compensatory anchors. This suggests that factors such as mobility and family values act as “threshold” requirements; they are foundational elements that the neural network recognizes as essential for predictive accuracy, even if they lack a consistent linear slope (Chang et al., 2012; Teo et al., 2015). Consequently, this hybrid approach proves that these predictors are vital to the decision-making process in a non-linear context, offering a novel perspective on PCA adoption that has received limited attention previously. These findings emphasize that for manufacturers and policymakers to develop truly effective solutions, they must look beyond linear drivers and integrate these “anchor” features into the core development schemes of parental control services.

The demographic profile of this study—primarily educated parents (65.9%) over the age of 30 (73.2%)—reflects a specific “professional parent” psychology in Saudi Arabia. Higher educational attainment often correlates with increased information security awareness, making technical mobility a baseline requirement for their digital workflows. Additionally, the mature age bracket typically exhibits a stronger protection motivation, viewing content control with greater urgency than younger cohorts. Consequently, for this demographic, the “fit” of a PCA is driven by a psychological preference for functional efficiency and risk mitigation. Although these traits characterize the most active consumer segment in the region, future research could explore younger or less tech-savvy populations to enhance the universality of these findings.

## 7 Contributions and implications

### 7.1 Theoretical contribution

The study’s findings contribute to the advancement of prior research on PCAs (Alelyani et al., 2019; Ghosh et al., 2018; Wisniewski et al., 2017; Mannan et al., 2020; Monteiro et al., 2017; Stoilova et al., 2024; Gnanasekaran and De Moor, 2025), whose conceptualizations of parental monitoring of children highlighted the critical role of technological solutions. Moreover, existing studies on the TTF model often focus on workplace productivity, leaving a gap in how “fit” is defined in domestic and protective settings. This study bridges that gap by redefining task characteristics to include socio-psychological factors like digital etiquette and cyberbullying. By doing so, it moves the theory beyond functional utility and into the realm of social-psychological mediation. Furthermore, although traditional literature relies on linear assumptions, this study addresses the gap by utilizing a hybrid SEM-ANN approach. This uncovers non-linear threshold effects—such as the role of mobility—which remain invisible in traditional SEM-only studies (Xin et al., 2025; Alharbi and Alkhalifah, 2025). This study provides a novel theoretical and empirical approach to the study of PCA adoption. To the best of our knowledge, this study is the first to employ TTF in the PCA context. Therefore, it contributes to both TTF and PCA research.

### 7.2 Practical contributions

For developers and policymakers, these findings emphasize that the “one-size-fits-all” approach to surveillance is insufficient. The significance of digital etiquette and cyberbullying indicates that parents are seeking tools that facilitate active mediation rather than passive monitoring. Practically, PCA manufacturers should prioritize features that provide social-contextual alerts and educational prompts over simple content filters. By addressing specific high-stakes tasks—such as mitigating digital harassment—developers can significantly enhance the perceived fit and subsequent adoption rates of their applications.

### 7.3 Social contributions

Socially, this research highlights a critical evolution in the family dynamic: the movement toward digital literacy and safety as a core parenting task. The support for parental mediation suggests that PCAs are being used as supportive technologies to foster open dialogue between parents and children regarding digital conduct (Stoilova et al., 2024). By identifying that parents prioritize etiquette and social protection, this study underscores the role of technology in maintaining community standards and protecting children’s psychological wellbeing in an increasingly connected society. This encourages a more holistic approach to child monitoring that balances technical protection with the development of a child’s digital citizenship.

## 8 Limitations and future work

First, the research was conducted within the KSA, a context in which parental supervision is deeply rooted in cultural norms. Consequently, the findings may vary significantly in nations with different cultural backgrounds or digital governance frameworks. Future research should undertake cross-cultural comparisons to determine whether the socio-psychological drivers of technology fit identified here remain consistent across diverse global demographics.

Second, the use of mall-intercept sampling may have introduced a socioeconomic selection bias (Memon et al., 2025). The sample was primarily limited to urban populations with the disposable income and leisure time to frequent such locations. As a result, the findings may not be fully representative of rural or lower-income demographics within KSA. To improve the generalizability and validity of the results, future studies should employ more diverse sampling techniques to include a broader range of socioeconomic backgrounds.

Third, although the ANN analysis successfully identified non-linear relationships, the “black box” nature of neural networks makes it difficult to pinpoint the exact directionality or causal mechanisms of these complex interactions. Subsequent studies could employ qualitative comparative analysis (fsQCA) to further explore the specific “configurations” of conditions—such as the combination of high cyberbullying concern and specific digital literacy levels—that lead to high adoption rates.

Finally, this study did not account for the moderating effect of age, as a generational comparison was beyond the current scope. Given that age is a well-established moderator in technology adoption literature, future research should investigate whether parental age or the child’s age significantly alters the perception of “fit” and subsequent behavioral intention. Conducting similar studies across various age groups will determine whether different parental generations prioritize PCA features differently.

## 9 Conclusion

The primary objective of this study was to evaluate how specific task characteristics and features influence parental adoption behavior regarding PCAs. By extending the TTF framework, this research examined the influence of mobility, digital etiquette, cyberbullying, child tracking, parental mediation, inappropriate content control, and family values on the behavioral intention to adopt PCAs. To our knowledge, this study represents the first application of the TTF model to analyze the adoption of child-monitoring technological solutions.

The research specifically analyzed how parents’ concerns regarding key features dictate the perceived “fit” of these solutions, offering insights into which features should be prioritized in development and how they should be promoted. According to the path coefficient findings from the PLS-SEM analysis, five hypotheses were supported, while three were rejected. However, the results from the supplemental ANN analysis confirmed the overarching predictive significance of all the investigated features, uncovering non-linear relationships that traditional linear models may overlook.

These findings suggest that parental adoption is driven by a complex alignment between high-stakes parenting tasks—such as mitigating cyberbullying and managing digital etiquette—and the technical capabilities of the apps. Consequently, developers and policymakers responsible for creating and piloting PCA solutions must comprehend these nuanced parental priorities. By facilitating and coordinating strategies that prioritize these robust and resourceful features, stakeholders can provide solutions that more effectively meet the safety needs of modern families.

## Data Availability

The raw data supporting the conclusions of this article will be made available by the authors, without undue reservation.

## Appendix A

Research items

**Table:**

Constructs	Code	Items	References
Mobility	MOB1	Using a parental control app is independent of time	Li et al. (2019), Zhou et al. (2010), and Mallat et al. (2009).
	MOB3	Using a parental control app is independent of place	
	MOB3	Using a parental control app is convenient because the phone is usually with me	
Child tracking	CTR1	The tracking feature is important for parents interested in the whereabouts of their child	Sukk and Siibak (2021)
	CTR2	The tracking feature helps me know the location of my child	
	CTR3	The tracking feature assures me that my child is in good hands	
Cyberbullying	CBB1	Digital citizenship education is a critical tool for combating cyberbullying	Zhong et al. (2021) and Zheng et al. (2020)
	CBB2	Fear of cyberbullying toward my child will encourage me to use a parental control app	
	CBB3	Apps with features to identify cyberbullying are important for me	
Digital etiquette	DET1	Guidelines and awareness to avoid violating privacy and infringement rules are important for me	Abulibdeh (2019)
	DET2	Digital etiquette is a critical tool for combating inappropriate behavior	
	DET3	I like apps that teach children specific examples of appropriate behavior or procedures when using technology	
Family values	FAV1	Positive family values are important for me	Wisniewski et al. (2017) and Badillo-Urquiola et al. (2020)
	FAV2	The use of value-sensitive design (VSD) in developing online safety apps will encourage me to use them	
	FAV3	Values such as obedience, honesty, discipline, trust, transparency, and openness play a significant role in my parenting	
Inappropriate online content	IOC1	I am concerned that inappropriate Internet content may endanger my child’s health or safety	Brito et al. (2017)
	IOC2	If my child is watching improper content, I encourage them to switch to something more educational	
	IOC3	If I see improper materials (most commonly violent) on my child’s phone, I remove them from the device	
	IOC4	I like apps that block or report inappropriate online content on my child’s phone	
Parental mediation	PAM1	I have concerns over my child’s safety	Ko et al. (2015) and Monteiro et al. (2017)
	PAM2	I will educate my child properly about limiting some device functions	
	PAM2	I will keep my child safe through parental mediation	
TTF	TTF1	In helping complete my parenting tasks, the functions of a parental control app are enough	Li et al. (2019) and Zhou et al. (2010)
	TTF2	In helping complete my parenting tasks, the functions of parental control apps are appropriate	
	TTF3	In general, the functions of a parental control app fully meet my needs when I am parenting	
Behavioral intention	BI1	I am likely to use a parental control app to monitor my child now or in the future	Venkatesh et al. (2003)
	BI2	I will try to use a parental control app to assist me in monitoring my child in my daily life	
	BI3	I intend to continue to use a parental control app frequently	
